# A Nonlinear Dynamic Approach Reveals a Long-Term Stroke Effect on Cerebral Blood Flow Regulation at Multiple Time Scales

**DOI:** 10.1371/journal.pcbi.1002601

**Published:** 2012-07-12

**Authors:** Kun Hu, Men-Tzung Lo, Chung-Kang Peng, Yanhui Liu, Vera Novak

**Affiliations:** 1 Division of Sleep Medicine, Brigham and Women's Hospital, Harvard Medical School, Boston, Massachusetts, United States of America; 2 Division of Gerontology, Beth Israel Deaconess Medical Center, Harvard Medical School, Boston, Massachusetts, United States of America; 3 Center for Dynamical Biomarkers and Translational Medicine, National Central University, Chungli, Taiwan; 4 Division of Interdisciplinary Medicine & Biotechnology and Margret & H.A. Rey Institute for Nonlinear Dynamics in Medicine, Beth Israel Deaconess Medical Center, Harvard Medical School, Boston, Massachusetts, United States of America; 5 Research Center for Adaptive Data Analysis, National Central University, Chungli, Taiwan; 6 DynaDx Corporation, Mountain View, California, United States of America; Johns Hopkins University, United States of America

## Abstract

Cerebral autoregulation (CA) is an important vascular control mechanism responsible for relatively stable cerebral blood flow despite changes of systemic blood pressure (BP). Impaired CA may leave brain tissue unprotected against potentially harmful effects of BP fluctuations. It is generally accepted that CA is less effective or even inactive at frequencies >∼0.1 Hz. Without any physiological foundation, this concept is based on studies that quantified the coupling between BP and cerebral blood flow velocity (BFV) using transfer function analysis. This traditional analysis assumes stationary oscillations with constant amplitude and period, and may be unreliable or even invalid for analysis of nonstationary BP and BFV signals. In this study we propose a novel computational tool for CA assessment that is based on nonlinear dynamic theory without the assumption of stationary signals. Using this method, we studied BP and BFV recordings collected from 39 patients with chronic ischemic infarctions and 40 age-matched non-stroke subjects during baseline resting conditions. The active CA function in non-stroke subjects was associated with an advanced phase in BFV oscillations compared to BP oscillations at frequencies from ∼0.02 to 0.38 Hz. The phase shift was reduced in stroke patients even at > = 6 months after stroke, and the reduction was consistent at all tested frequencies and in both stroke and non-stroke hemispheres. These results provide strong evidence that CA may be active in a much wider frequency region than previously believed and that the altered multiscale CA in different vascular territories following stroke may have important clinical implications for post-stroke recovery. Moreover, the stroke effects on multiscale cerebral blood flow regulation could not be detected by transfer function analysis, suggesting that nonlinear approaches without the assumption of stationarity are more sensitive for the assessment of the coupling of nonstationary physiological signals.

## Author Summary

Cerebral autoregulation is an important mechanism that regulates blood supply to brain tissue to match metabolic demands during daily activities. Impaired cerebral autoregulation increases the dependence of cerebral blood flow on systemic blood pressure, and is associated with fatal outcomes in patients after brain injury and acute ischemic stroke. Reliable and noninvasive assessment of cerebral autoregulation is still a major challenge in medical diagnostics and clinic studies, mainly because blood pressure and flow are intrinsically nonstationary (possessing complex oscillations/fluctuations with varying amplitude and frequency) while traditional methods for assessment of the pressure-flow dependence assume stationary signals. We propose a new computational technique that is based on nonlinear theories without the assumption of stationary signals. This technique allows us to detect the degradation of cerebral autoregulation in patients with mild ischemic stroke even at >6 months after the insult. The degradation was present in both stroke and non-stroke sides and was accompanied by an altered pressure-flow interaction over a wide range of frequencies from 0.02–0.38 Hz. Our results challenges the traditionally accepted functional region of autoregulation (<∼0.1 Hz). The observed long-term influences of stroke highlight the importance of reliable monitoring of cerebral blood flow regulation for the management and daily care of stroke patients.

## Introduction

Cerebral blood flow (CBF) is regulated to provide adequate blood supply to brain. One of the important CBF control mechanisms is cerebral autoregulation (CA) [1]. Involving dilation and constriction of cerebral arterioles through myogenic and neurogenic regulation, CA allows to maintain relatively stable CBF despite changes of systemic blood pressure (BP) [2]–[7]. Impaired CA leads to more dependence of CBF on BP, leaving brain tissue unprotected against the potentially harmful effects of BP fluctuations, as demonstrated in cerebromicrovascular disease associated with diabetes [8]–[10], or after ischemic stroke [11]–[14] and brain injury [15]–[17].

A widely accepted concept of CBF regulation is that CA is less effective or even inactive at high frequencies (>∼0.1 Hz) or at small time scales (<∼10 seconds), thus leading to a passive dependence of CBF on BP at small time scales [18]. Many studies supported this concept [19]–[22] but all were exclusively based on transfer function analysis (TFA) that utilizes the Fourier transform to quantify the relationship between BP and cerebral blood flow velocity (BFV; recorded by transcranial Doppler) [18]. The TFA assumes stationary signals while physiological signals including BP and BFV are highly nonstationary, displaying complex fluctuations at different time scales with varying amplitude and period even during baseline conditions [23], [24]. Moreover, TFA assumes a linear relationship between two signals while it is well known that CA leads to a nonlinear pressure-flow interaction [18]. Thus, TFA may render unreliable or even misleading results [25], [26]. To better understand CBF regulation at different time scales or frequencies, we introduce a new analytical tool termed intrinsic multiscale pressure-flow analysis (IMPFA) that has no assumptions of linearity and stationarity. Based on the empirical decomposition analysis [27], [28], this method extracts intrinsic oscillations of BFV and BP at multiple time scales and provides a pressure-flow spectrum to quantify dynamic BFV-BP interaction (see Methods). Using this new method, we aimed to establish the multiscale relationship between spontaneous BP fluctuations and BFV fluctuations in old adults, and to determine the long-term effects of ischemic stroke on CBF regulation.

Normal CA function is characterized by a faster recovery of the BFV than BP (i.e., BFV has advanced phases compared to BP) and can be estimated by specific phase shifts between BP and BFV oscillations [29], [30]. We hypothesize that autoregulation is a continuous process operating over a wide range of time scales (from a few heart beats to about one minute), and that the multiscale autoregulation can be quantitatively assessed from the phase shift between baseline BFV and BP fluctuations. We further hypothesize that stroke leads to permanent CA impairment and affects BFV-BP phase relationship at multiple time scales.

## Results

### BFV and BP fluctuations are complex and BFV-BP phase shift was frequency dependent

**Figure 1:**
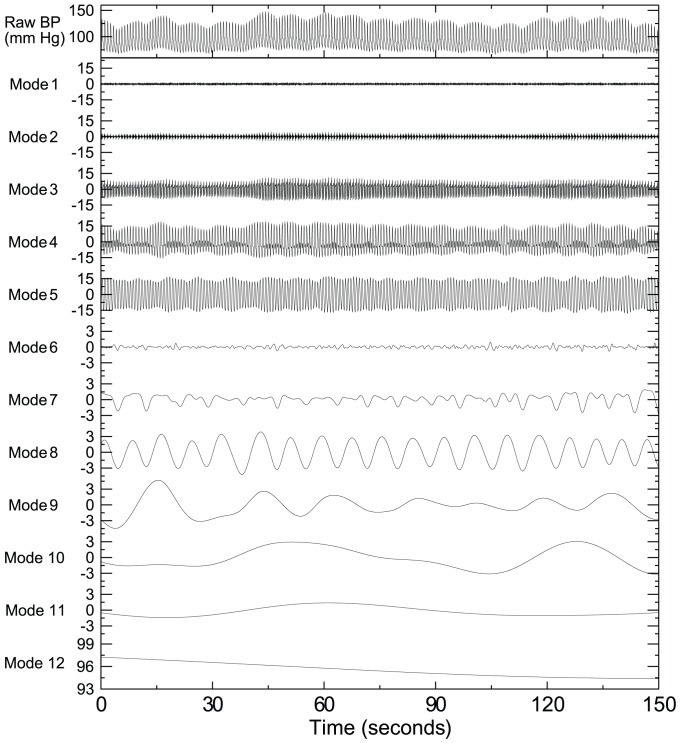
Demonstration of empirical mode decomposition. A blood pressure (BP) recording was collected from finger plethysmography in a non-stroke subject during supine rest condition (Top panel). Decomposed oscillatory components of the signal are shown in the followed panels. Each component consists of oscillations within a narrow frequency band. Note that the amplitude and period of oscillations in each component were not constant, indicating nonstationarity in the BP signal.

**Figure 2:**
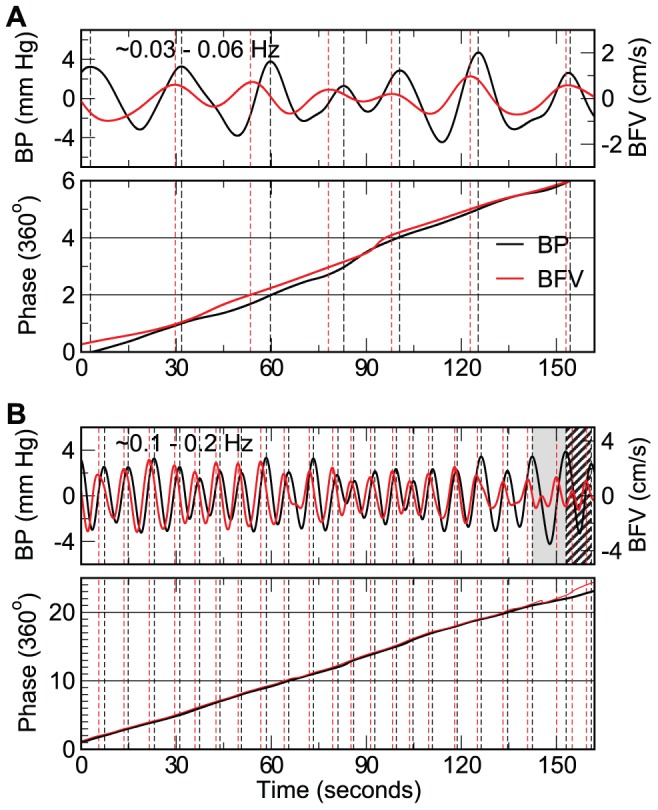
Phase interaction between intrinsic oscillations of cerebral blood flow velocity (BFV) and blood pressure (BP) at different time scales or frequencies. Examples of extracted BP and BFV components, and their instantaneous phases at ∼0.03–0.06 Hz (A) and ∼0.1–0.2 Hz (B). Oscillatory components were extracted from original BP and BFV signals using the empirical decomposition method and their instantaneous phases were obtained using the Hilbert transform. Individual cycles (separated by dashed lines) are identified based on instantaneous phases and each cycle corresponds to a phase increase of 360°. In each extracted component, oscillations are not stationary with varying amplitude and period. Generally, the phase of BFV oscillations was advanced compared to the phase of the corresponding BP oscillations. Data in the last two BP cycles in B were excluded because either (i) instantaneous phases BFV decreased as characterized by an extra oscillation under the zero line (the grey-highlighted cycle; see Criterion 1); or (ii) the BP period was significantly greater (>1.5 times) compared to the BFV period (the hatched cycle; see Criterion 3).

**Figure 3:**
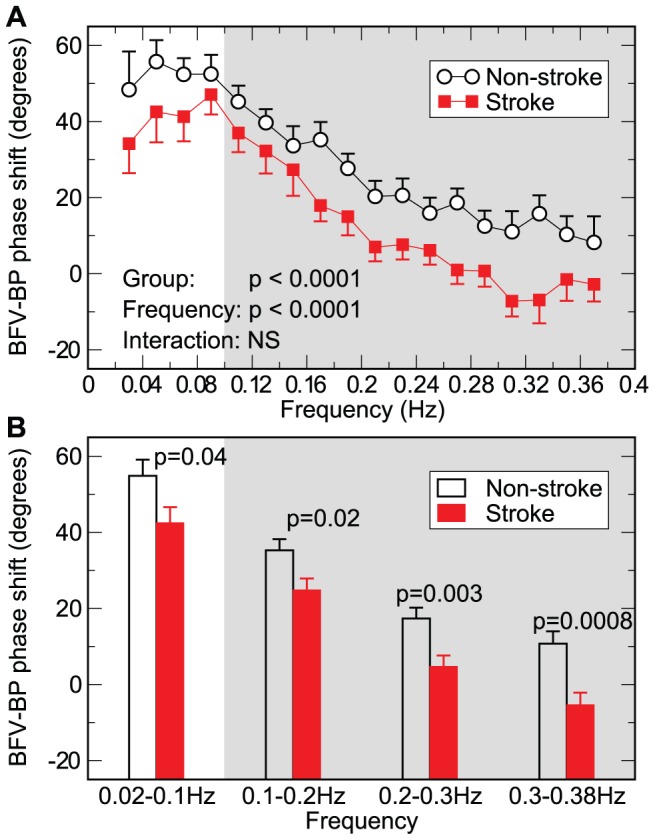
Group average of phase shift between BP and BFV oscillations extracted by the empirical decomposition. Results were presented (***A***) in 18 frequency bins with the same size of 0.02 Hz from 0.02 to 0.38 Hz and (**B**) in 4 frequency bins (0.02–0.1 Hz, 0.1–0.2 Hz, 0.2–0.3 Hz, and 0.3–0.38 Hz). Data are presented as Mean±SE, where Mean was obtained by averaging the individual means for non-stroke (or left) and right (stroke) sides and SE indicates between-subject error. Shown are P values for group difference, frequency effect, and the interaction between group and frequency. Here “NS” indicates P>0.1. The gray region indicates the frequency range (>0.1 Hz) where cerebral autoregulation was often considered to be less or even not active.

Both BFV and BP signals showed complex fluctuations across a wide range of time scales. Figure 1 showed all intrinsic oscillatory components of a BP signal that were obtained in the first step of the proposed IMPFA using the empirical mode decomposition (see Methods) [27], [28]. Figure 2 shows two of these oscillatory components and the corresponding BFV components in the frequency range of ∼0.03–0.06 Hz (corresponding to cycle length of ∼16.7–30 seconds) and ∼0.1–0.2 Hz (corresponding to cycle length of ∼5–10 seconds), respectively. The amplitude and period of oscillations were highly variable among different cycles even in the same component (see Text S1; Figure S1). The variability may be due to nonstationary properties of the BP and BFV signals as well as noise and artifacts in the recordings (see Methods). In the non-stroke group, the mean BFV-BP phase shift was positive for all tested time scales. Such phase shift would be expected during an active cerebrovascular regulation that leads to a faster recovery in BFV compared to BP fluctuations (see simulation results below). The value of BFV-BP phase shift was generally larger at low frequencies (or large time scales) and smaller at high frequencies (mixed model ANOVA p<0.0001) (Figure 3A), e.g., BFV-BP phase shift in the non-stroke group decreased from 54.8±4.3° (Mean±SE) at 0.02–0.1 Hz to 10.7±3.2° at 0.3–0.38 Hz (Figure 3B). Similar frequency dependence was also observed in the stroke group, i.e., smaller phase shift at higher frequencies (p<0.0001). We note that the phase shift value in non-stroke group remained relatively constant within the frequency range of 0.02–0.1 Hz while the stroke group showed a maximal phase shift at ∼0.09 Hz (post hoc p<0.05).

### BFV-BP phase shift was reduced in stroke subjects over a wide range of frequencies

BFV-BP phase shift was consistently smaller in the stroke group compared to the non-stroke group over the entire examined frequency range of 0.02–0.38 Hz (p<0.0001) (Figure 3A). For instance, at 0.02–0.1 Hz, BFV-BP phase shift in the stroke group was 42.5±4.1° (SE) (∼12° smaller than the value in the non-stroke group); at 0.3–0.38 Hz, BFV-BP phase shift was −5.4±3.2° (16° smaller than the value in non-stroke group) which was statistically indistinguishable from zero (Wilcoxon signed-rank test p>0.35). The mixed mode ANOVA indicated that there was no significant interaction between effects of group and frequency (p>0.8), i.e., the group difference was not significantly dependent on frequency. Repeating the same statistical analysis at four separated frequency bins (0.02–0.1 Hz, 0.1–0.2 Hz, 0.2–0.3 Hz, 0.3–0.38 Hz) confirmed reduced phase shift in stroke patients at all frequencies (Figure 3B). We note that the p value for the group difference was smaller for bins at higher frequencies (smaller p value indicate more significant group difference). This difference in p value might be partially explained by the fact that there were generally more cycles for a better estimate of mean BFV-BP phase shift at higher frequencies.

In this case-control study, age, sex, BMI, mean BP, and baseline CO_2_ were matched between the non-stroke and stroke groups (Table 1). Thus, the observed group difference in BFV-BP phase shift was independent of these variables. To test whether the reduced BFV-BP phase shift in the stroke group was associated with the changes in cerebrovascular resistance and/or CO_2_ vasoreactivity (Table 1), we repeated the statistical analysis with including these variables as covariates in the mixed models. We found that neither variable had significant influences on BFV-BP phase shift (p>0.12 for both variables) while the effects of group and frequency persisted. Using the similar approach, we found that the observed group difference in BFV-BP phase shift was also independent of mean BP (p>0.7), hypertension or normtension (p>0.2), mean BFV (p>0.3), and mean heart rate (p>0.08). Moreover, the difference between the stroke and non-stroke groups remained (p<0.0001) when only normotensive subjects (13 stroke and 23 non-stroke subjects) were included in the analysis.

**Table 1:** Demographic characteristics for non-stroke and stroke groups.

	Non-stroke (N = 40)	Stroke(N = 39)	p value
Age (range) (years)	68.0±1.0 (51–80)	64.6±1.4 (50–80)	NS
Sex Male/Female	17/23	20/19	NS
Body mass index	25.7±0.6	27.2±0.7	NS
HTN	17	26	NS
Mean blood pressure (mmHg)	84.0±1.5	85.8±1.6	NS
Heart rate (bpm)	65.0±1.4	69.7±1.4	0.022
CO_2_ level (mmHg)	36.5±0.4	35.2±0.8	NS
Cerebral blood flow velocity (cm/s)			0.0036*
Stroke or right side	46.7±3.2	37.0±2.6	
Non-stroke or left side	48.0±2.3	37.8±3.2	
Cerebrovascular resistance (mm Hg s/cm)			0.0006*
Stroke or right side	2.1±0.2	2.8±0.3	
Non-stroke or left side	1.9±0.1	3.0±0.3	
CO_2_ vasoreactivity			0.034*
Stroke or right side	1.2±0.2	0.8±0.2	
Non-stroke or left side	1.4±0.2	0.8±0.2	

Data are presented as Mean±SE. All p values are for comparisons between the non-stroke and stroke groups.

*indicates no difference between stroke and non-stroke sides.

**Figure 4:**
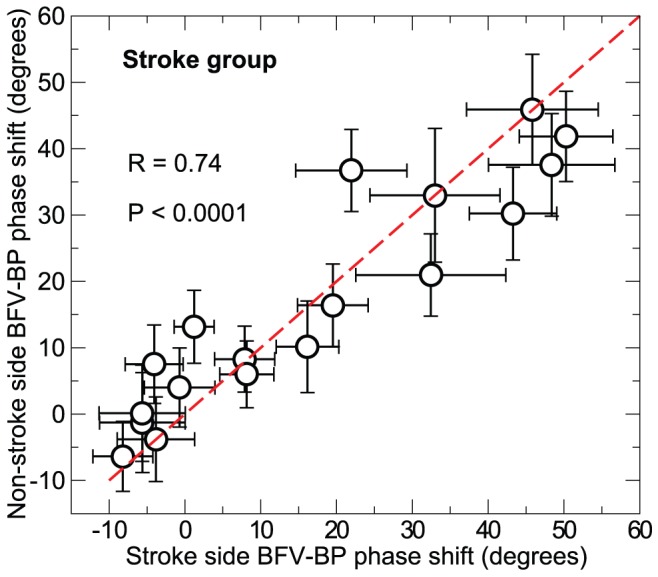
Comparison of phase shifts between the stroke and non-stroke sides. Data of stroke subjects were presented. Each data point indicates the group means of BFV-BP phase shifts for stroke side (x axis) and non-stroke side (y axis) in each of 18 frequency bins from 0.02 to 0.38 Hz. Error bars indicate standard error between subjects. A strong correlation between the phase shifts in stroke and non-stroke sides was observed based on a linear regression analysis (R = 0.74, p<0.0001), in which individual data (instead of group means) were used while the effect of frequency was accounted.

Within the stroke subjects, BFV-BP phase shifts were not different between the stroke and non-stroke sides at all tested frequencies (p>0.7). Indeed phase shifts of the stroke and non-stroke sides were highly correlated (p<0.0001, r = 0.74; Figure 4). These observations remained the same for 13 stroke subjects without hypertension (non-significant side effect: p>0.8; side correlation: p<0.0001, r = 0.85). In addition, BFV-BP phase shifts at all tested frequencies were not significantly correlated with the time period after the stroke for both stroke (p>0.3) and non-stroke sides (p>0.8).

### Larger BFV-BP phase shift indicates faster response in cerebrovascular resistance

**Figure 5:**
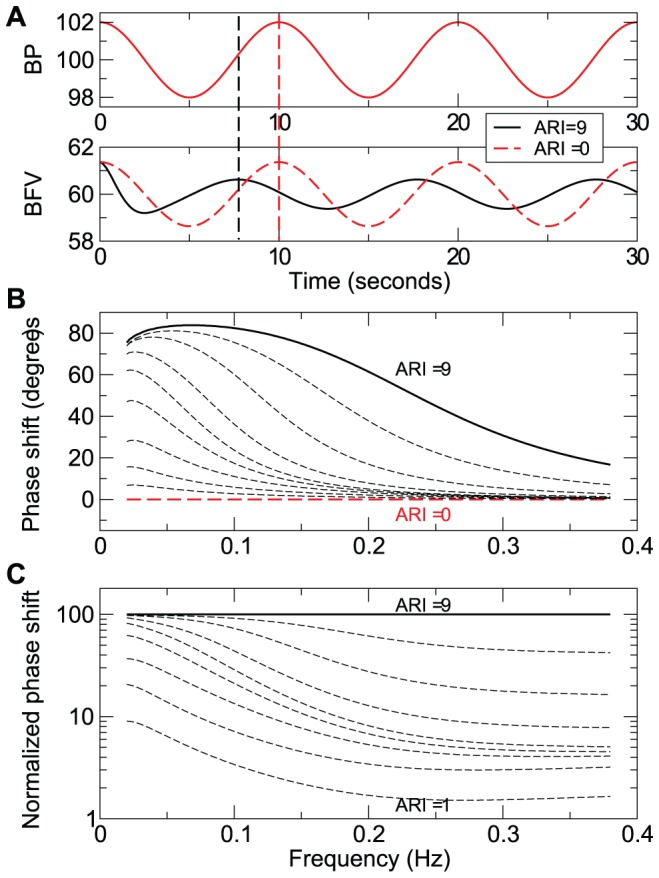
Frequency-dependent phase shift between BP and BFV oscillations in the Aaslid-Tiecks model. (**A**) BP oscillations at certain frequency induced oscillations in BFV at the same frequency. In the Aaslid-Tiecks model [56], cerebral autoregulation is estimated by a dynamic autoregulation index (ARI) that ranges from 0 (absence of cerebral autoregulation) to 9 (best autoregulation). When cerebral autoregulation is absent (ARI = 0), there is no phase shift between BP and BFV oscillations, i.e., the locations of BFV and BP peaks coincide. (**B**) BFV-BP phase shift at different frequencies for different autoregulation (ARI = 0 to 9 from bottom to top). (**C**) Normalized BFV-BP phase shift as percentages of values of ARI = 9. Curves from bottom to top correspond to ARI = 1 to 9, respectively. Note that y axis was in log scale. Results in B and C showed that BFV-BP phase shift decreased with increasing frequency and that phase shift is larger for larger ARI at all tested frequencies (0.02–0.38 Hz).

To demonstrate that BFV-BP phase shift is an autoregulation measure, we examined BFV changes in response to oscillatory BP fluctuations utilizing the Aaslid-Tiecks model [29], [31]. This model was originally used to simulate CBF or BFV recovery in response to sudden BP drop for different degree of cerebral blood flow regulation that is characterized by an autoregulation index (ARI). Ranging from 0 to 9, ARI indicates how quickly resistance can be adjusted and BFV can be restored in response to BP change, i.e., ARI = 0 for no autoregulation or no recovery of BFV and ARI = 9 for best autoregulation [29], [31]. In this study, we used a sinusoidal waveform to simulate BP oscillations (Figure 5). As expected, the Aaslid-Tiecks model showed that BP oscillations led to BFV oscillations with a sinusoidal waveform at the same frequency of BP (Figure 5A). BFV-BP phase shift was zero when ARI = 0 (red lines in Figures 5A), indicating a passive dependence of BFV on BP. At a fixed frequency of BP oscillations, BFV-BP phase shift increased monotonically when ARI increased from 0 to 9 (Figure 5B, 5C). Such a relationship between BFV-BP phase shift and ARI remained at all tested frequencies from 0.02–0.38 Hz (Figure 5). These results are consistent with the well-accepted belief that a large BFV-BP phase shift indicates a better autoregulatory function with quicker resistance adjustment.

The simulations also showed that BFV-BP phase shift for any ARI>0 was generally larger at lower frequencies and become smaller at higher frequencies (Figure 5). The similar frequency dependence was also observed in human data (Figure 3). Thus, it is important to specify frequency of BP/BFV oscillations when comparing BFV-BP phase shift for assessment of differences or changes in CA. In addition, this frequency-dependent relationship is a key feature indicating that BFV-BP phase shift is not caused by a simple time delay between BP and BFV (see Text S2; Figure S2).

## Discussion

Most studies of cerebral autoregulation in health and disease were focused on frequencies ≤0.1 Hz [18]–[22]. It is often presumed that the BFV-BP relationship at smaller time scales is beyond the range of autoregulation. Using the IMPFA, we demonstrated that the phase of BFV oscillations is advanced compared to BP oscillations at 0.02–0.38 Hz, indicating an active cerebral blood flow regulation at multiple time scales (∼2.6–50 seconds). Ischemic stroke affected this multiscale regulation even at ≥6 months after stroke insults, leading to more passive dependence of BFV on BP that is characterized by consistent reduction of BFV-BP phase shift at all tested frequencies. In addition, this long-term stroke effect on cerebral blood flow regulation was similar in the infarcted and non-infarcted hemispheres. These findings raise the possibility that dynamic CA may be engaging in cerebral blood flow regulation over a wide range of time scales instead of being limited to a narrow band at large time scales, thus providing a fast perfusion control.

### Physiological meaning of BFV-BP phase shift at different time scales

There is clear evidence that perfusion is only *relatively* stable and BP fluctuations can induce CBF variations at different frequencies. For instance, Claassen et al. showed that repeated squat-stand maneuvers could induce oscillations at a designed frequency in both BP and BFV in healthy young individuals with normal autoregulation [32]; and we also showed BFV during baseline conditions possessed oscillations that matched to BP oscillations over a wide range of time scales (0.02–0.38 Hz; Figures 1–2). Indeed, these BFV variations in response to BP fluctuations can provide important information about cerebrovascular control system. In this study, we focused on one of the useful biomarkers derived from BFV and BP variations, namely BFV-BP phase shift. This measure can reflect dynamic CBF regulation via adjustment of cerebrovascular resistance (Figure 5) although it can also be affected by other vascular properties such as absolute levels of baseline cerebrovascular resistance and compliance [33].

The physiological understanding of CBF (or BFV) regulation at different time scales is still debated and a high-pass filter-cybernetic model is often used to describe the coupling between BP and CBF/BFV [34]. This model predicts that a very slow oscillation in BP (frequency approaching zero) will generate an oscillation in BFV with very small amplitude and an advanced phase close to 90°, while a fast oscillation in BP will be completely transmitted to a BFV oscillation with phase lag close to zero. This frequency-dependent feature is well demonstrated in our human data (Figure 3) and our simulations using the Aaslid-Tiecks model (Figure 5), as well as in previous studies using the TFA [5], [32], [34], [35].

It is important to note that the high-pass model did not suggest a particular cutoff frequency or time scale of CA function. Many CA studies using TFA did consider the BFV-BP interaction over a wide range of frequencies including >0.1 Hz [5], [19], [21], [32], [33], [36]. However, most of these studies refute that BFV-BP relationship at frequencies >0.1 Hz is useful in term of detecting physiological and pathological changes in cerebrovascular system [18]. These TFA-based studies might underlie the widely accepted concept of the active CA region at low frequencies. Using the proposed IMPFA, we showed in this study that the effect of stroke on the BFV-BP interaction persists at high frequencies (0.1–0.38 Hz) as well as at low frequencies (∼0.02–0.1 Hz), leading to a reduction of BFV-BP phase shift at multiple time scales. Additionally, our simulation results based on the Aaslid-Tiecks model confirmed that the association between BFV-BP phase shift and the degree of autoregulation remained at all tested frequencies from 0.02–0.38 Hz (Figure 5). These results indicate that the BFV-BP phase shift at high frequencies may also provide insights into CBF regulation.

A possible concern on the multiscale flow-pressure coupling is whether small phase shift at high frequencies was simply caused by a time delay between BFV and BP recordings. Since BFV was measured from arteries in brain and BP was measured from finger, the pulse transit time could be different for the two locations. The possible time delay would artificially induce certain BFV-BP phase shift that contains not much physiological information. This is unlikely because the phase shift in both simulation and experimental data became smaller at higher frequencies while a simple time delay would predict a larger phase shift at higher frequencies (see Text S2; Figure S2). However, a time delay may still contribute to BFV-BP phase shift, especially at frequencies >0.3 Hz in control subjects (Figure S3), leading to an overestimation of the phase shift. Such time-delay effect may potentially contribute to the observed reduction of BFV-BP phase shift in stroke subjects at the high frequencies since stiffer arteries in these populations can lead to a reduced time delay. Furthermore, the frequency-dependent pressure-flow relationship can also be described by a mechanical model without active control of cerebrovascular resistance — Windkessel model [33], [37], [38], in which the cerebral arterial bed is composed of both resistance and compliance elements. In this model, the compliance element can lead to phase advance of flow oscillations, and the magnitude of the resultant phase shift is smaller at higher frequencies. Therefore, further studies are required to formally determine how the mechanical properties of vasculature (vascular compliance and stiffness) contribute to pressure-flow phase shift and its reduction in stroke, especially at high frequencies.

To ensure a physiologically meaningful and reliable estimate of BFV-BP phase shift, we only studied frequencies up to 0.38 Hz. This choice was mainly based on the following two considerations. There were not enough matched BFV-BP oscillations at frequencies between ∼0.38 Hz and ∼1 Hz because there were not enough BP oscillatory components over the frequency range. In addition, the potential time-delay effect as discussed above would have stronger effect on the phase shift estimation at >0.38 Hz. For instance, the estimated time delay between BP and BFV recordings in control subjects was generally ∼50 ms (see Text S2 and Figure S3), which would lead to an artificial phase shift of ∼7° at 0.38 Hz and ∼18° at 1 Hz.

### Physiological mechanisms underlying multiscale BFV-BP coupling

Multiple control mechanisms are involved in the CBF regulation from the cellular level to the neurovascular unit to regional blood flow in main vascular territories. These feedback mechanisms, including myogenic [2], metabolic [3], [4], [39], [40], endothelial [41], and neurogenic regulations [5], [6], can operate at multiple time scales from seconds to minutes [42]. For instance, stretch of vascular muscles resulting from changes of intravascular pressure can induce vasodilatation or constriction through myogenic [2] and endothelial [7] responses within a few heartbeats; cholinergic dilation of cerebral blood vessels is also a rapid process that is engaged within neurovascular unit comprising of a neuron, astrocyte and a microvessel [6]; and sympathetic modulation is involved in modulating overall vascular tone at larger time scales (>20 seconds) [5]. These mechanisms are integrated within the CA process to accommodate and redistribute CBF locally and regionally in response to changes of neuronal activity, variations of arterial BP, and other physiological stimuli. Future studies are required to test whether these complex mechanisms are capable of affecting BFV-BP coupling across the wide range of frequencies from 0.02 to 0.38 Hz or other vascular mechanisms (different from cerebral autoregulation) are responsible for flow-pressure phase interaction at high frequencies (>0.1 Hz).

### Influences of stroke on cerebrovascular system

There is accumulating evidence that influences of stroke on the cerebrovascular system evolve in time and space and that they may extend to regions distant form infarct site [43]–[46]. Our recent study supported this notion, showing impaired vascular reactivity in larger areas of brain including vascular territories that are distant from the infracted site and were not damaged by acute infarction [47]. Such long-term and dynamic effects of stroke on cerebrovascular system may explain our finding of similar degradation of CBF regulation in both stroke and non-stroke sides in the stroke patients.

Vascular changes associated with other cerebromicrovascular diseases than stroke such as hypertension and diabetes can also lead to impaired autoregulation [8]–[10], [14] and increase risk for stroke [48]. Thus, it is possible that the observed impaired autoregulation in both stroke and non-stroke sides may be caused by certain vascular complication other than stroke that may precede stroke onset or may be one of the causes of ischemic stroke. To explore such possibility, we have checked many vascular measures in this study including heart rate, blood pressure, mean BFV, cerebrovascular resistance, and CO_2_ reactivity. Though many of these variables showed significant differences between the stroke and non-stroke groups, none of them could account for the observed group difference in BFV-BP phase shift. For instance, we showed that the group difference persisted when excluding hypertensive subjects, suggesting that the group difference was not caused by the possible effect of hypertension or antihypertensive medications. Note that such results did not exclude the possible effects of antihypertensive drugs on CBF regulation via their influences on the autonomous nervous system, which shall be addressed in future studies. Overall, our results strongly suggest that the global degradation of autoregulation in the stroke group reflects more likely the long-term effect of stroke. However, in order to formally prove or refute this hypothesized mechanism, it is necessary to examine autoregulation before, immediately following and after stroke for the same subjects. Such data are not available in this study and future longitudinal and prospective studies are warranted.

We note that the stroke subjects were studies at quite different time after stroke insults (i.e., 0.5–30.9 years). This is not an ideal approach considering the fact that the time course of the long-term effect of stroke on cerebral blood flow regulation is not clear. In this study, we did not find significant association between BFV-BP phase shift and time after stroke (stroke side: p>0.3, non-stroke side: p>0.8). These preliminary results suggest that the observed impairment of cerebral blood flow regulation may occur within the 6 months following stroke insults. Since this is a pilot study with a small sample size, future large-scale longitudinal and prospective studies are needed to determine the long-term impact of stroke on cerebral blood flow regulation.

### Nonlinear approaches for CA assessment

Most of previous studies of CA at different time scales (frequencies) utilized the TFA [18]. This traditional approach is based on the Fourier transform, assuming that BP and BFV signals are stationary and are composed of superimposed sinusoidal oscillations of constant amplitude and period at a pre-determined frequency range [26]. Recently, it has been realized that physiological signals are intrinsically nonstationary, and that traditional analysis with the stationary assumption may be unreliable or even invalid [25], [26]. In addition, the TFA assumes a linear relationship between two signals and this assumption is often verified or refuted by checking one TFA-derived measure, namely coherence that ranges from 0 to 1. The mean TFA coherence for the BFV-BP relationship is smaller than ∼0.75 at all frequencies <0.5 Hz and even smaller at frequencies <0.1 Hz (close to or less than 0.5) (see Text S3; Figure S4). The low coherence at <0.1 Hz is believed to reflect cerebral autoregulation that leads to a nonlinear BFV and BP interaction. Such a belief would indicate that TFA-derived BFV-BP phase shift and gain at low frequencies are not valid although these TFA measures have been widely used to assess cerebral autoregulation and its change under physiological and pathological conditions [5], [19], [21], [32], [33]. Therefore, the interpretation of TFA results should deserve more careful considerations and further theoretical studies shall be conducted to resolve the current contradiction in the interpretations of TFA results.

To better handle nonstationary signals, the multimodal pressure-flow analysis (MMPF) was introduced and has been successfully applied to identify altered CA in hypertension, diabetes, stroke, and brain injury [8], [14], [17], [49]. Though the MMPF is also based on the empirical mode decomposition, several notable concerns remain for this analysis. First only a single BP mode and its corresponding BFV mode were selected and used to quantify the BFV-BP phase interaction while all other components of BP and BFV are ignored. Thus, the rich multiscale dynamic information in BFV and BP fluctuations is not fully examined in the MMPF. Second, the MMPF estimates BFV-BP phase shift by averaging all oscillatory cycles that could have different frequencies due to the intrinsic nonstationary feature of physiological signals (Figure 2 and Figure S1). Depending on the cycle frequencies in the selected mode, the estimated mean phase shift can vary because it depends on frequency (Figure 3). Moreover, artifacts caused by either data acquisition (missing data or outliers) or nonstationarity in the data often exist and contaminate each oscillatory component extracted by the EMD, affecting the performance of the MMPF [26]. Thus, the performance of the MMPF is limited because BFV-BP phase shift is frequency dependent (Figure 3) and nonstationarity is an intrinsic property of many physiological signals (Figure 2).

The proposed IMPFA overcomes many limitations of the MMPF and TFA by examining the phase shift of intrinsic cycle-by-cycle BFV-BP oscillations at different time scales. As compared to the MMPF, the IMPFA uses a spectrum to describe frequency-dependent phase interaction between BP and BFV oscillations, thus providing more dynamic information in a more accurate manner. Moreover, the IMPFA is designed to better account for nonstationarities and noise in BP and BFV recordings by filtering out data without matched BFV-BP cycles. We also performed the TFA analysis in this study but the TFA-derived BFV-BP phase shift did not reveal any stroke effect (p>0.07 for both sides) (see Text S3; Figure S4). This might be due to influences of nonstationarities and noise in BP and BFV signals which can introduce significant variations and random errors in the TFA results (see Text S3) [26].

In addition to the EMD-based approaches, many sophistical analyses derived from modern concepts and techniques of nonlinear dynamics such as synchronization, wavelet transform, and adaptive filtering have been gradually applied to biological and physiological research [50]–[52]. The current and previous findings strongly suggest that these nonlinear approaches without the assumption of stationarity are more suitable for the assessment of complex physiological interactions including the BP and BFV coupling.

### Conclusion and future directions

In summary, we demonstrated a multiscale regulation in cerebral blood flow during supine resting conditions and showed a long-term effect of stroke that alters the regulation in both hemispheres, thus compromising the ability to counteract perturbations imposed on brain tissue during perfusion pressure fluctuations. With impaired cerebral blood flow regulation, pressure fluctuations are transmitted into cerebral vasculature, exposing brain tissue to potential harmful variations of perfusion and limiting the delivery of blood flow to areas of increased metabolic demands. Thus, our findings highlight the potential importance and benefit of reliable and non-invasive CBF regulation monitoring for the management and daily care of stroke patients. These findings also provide new insights into cerebral blood flow regulation and also raise a new challenge to the modeling of the cerebral autoregulation function. Different control mechanisms such as myogenic, metabolic, and neurogenic controls are involved in the CBF regulation. Future studies are needed to test whether each of these control mechanisms contributes to flow-pressure coupling in a specific time scale range or at all time scales, and to determine which mechanisms affected by stroke are responsible for the altered multiscale cerebral blood flow regulation in these patients.

## Methods

### Ethics statement

All data were previously collected in the Syncope and Falls in the Elderly Laboratory at Beth Israel Deaconess Medical Center (BIDMC). All participants provided informed consent and research protocols were approved by the local Institutional Review Board.

### Subjects

To test our hypotheses, we studied 79 participants (50–85 years old) with 39 stroke patients and 40 age- and sex-matched non-stroke subjects. Subjects were recruited from the community-living older people via advertisement in local newspapers. All subjects were screened with a medical history, physical examination, standard battery of autonomic tests and routine blood and urine chemistries. All Stroke subjects had chronic large artery hemispheric MCA infarcts documented on MRI or CT during the acute phase. Neurological and functional status of stroke patients was assessed by NIHSS (mean±SE: 2.6±0.4) and a Modified Rankin Scale (1.1±0.2; <4 for all patients, indicating ability to walk). Studies were conducted at 0.5–30.9 years (mean = 6.1 years) after stroke when these patient were clinically stable. The 40 non-stroke subjects had no clinical history of stroke, no known carotid stenosis, and no focal deficits on neurological examination. Twenty-six of the stroke patients and 17 of the non-stroke subjects had hypertension that was defined as use of antihypertensive medications or systolic BP >140 mm Hg or diastolic BP>85 mm Hg on 24 hour BP monitoring. Antihypertensive medications were tapered and withdrawn for 3 days prior to the study with home BP monitoring. We excluded subjects with intracranial or subarachnoid hemorrhage on MRI or CT, diabetes mellitus, clinically significant arrhythmias, uncontrolled hypertension (systolic BP>180 mm Hg and/or diastolic BP>100 mm Hg; or subjects taking ≥3 antihypertensives), morbid obesity, contralateral carotid stenosis >50% cases (for stroke patients), or any contraindications to MRI. There were no significant differences in age, body mass index (BMI), mean BP, and CO_2_ level between the stroke and non-stroke groups (Table 1). Baseline heart rate was higher and the mean BFV was lower (for both stroke and non-stroke sides) in the stroke group compared to the non-stroke group. Within the stroke subjects, mean BFV did not show significant difference between the stroke and non-stroke sides (p>0.6).

### Experimental procedure

The experiment was performed between ∼10AM–11:30AM (at least 2 hours after the last meal) after subjects stayed overnight in the inpatient room of the Harvard Clinical and Translational Science Center at BIDMC. Before the test, Subjects were resting comfortably in supine position in a quiet environment for at least 20 minutes. Then data were collected for at least 5 minutes during a baseline condition when subjects remained awake and relaxed in the horizontal and supine position. Following the baseline, CO_2_ vasoreactivity was assessed by performing a 3-minute hyperventilation test and a 3-minute test of rebreathing air in a bag with 5% CO_2_.

### Data acquisition

Changes in systemic BP were continuously assessed by measuring beat-to-beat BP waveforms from a finger using a Finapres device (Ohmeda Monitoring Systems, Englewood CO) [18]. BFV was simultaneously measured from left and right middle cerebral arteries using transcranial Doppler ultrasonography system (PMD150 Spencer Technologies, Inc., WA). Doppler probes were positioned to achieve maximal BFV, and stabilized using a 3-D holder. During the data collection, subjects were instructed to minimize movement and to breathe at their normal respiratory frequency. Thus, the diameter of the insonated artery, insonation angle and plasma hematocrit remained relatively constant such that changes in BFV reflect changes in CBF, and the BFV-BP phase relationship can represent the CBF-BP phase interaction in the territory of the insonated vessel [53]–[55]. The electrocardiogram was measured from a modified standard lead II using a Spacelab Monitor (SpaceLab Medical Inc., Issaquah, WA). End-tidal CO_2_ values were also recorded from the face mask (Capnomac Ultima, Ohmeda Monitoring Systems, Englewood, CO). Data were continuously recorded at a sampling frequency of 500 Hz and was re-sampled to 50 Hz for data analysis.

Note that the time course of the CO_2_ change in cerebral arteries during breathing tests could vary considerably between subjects, depending on the lung function and blood gas transport mechanisms of individuals. To account for such individual difference, we selected a 30-second window during the hyperventilation test when BFV reached minimum and a 30-second window during the CO_2_ rebreathing test when BFV reached the maximum. The mean BFV levels in the two 30-seconds windows were used to calculate the CO_2_ vasoreactivity.

### Intrinsic multiscale pressure-flow analysis (IMPFA)

To quantify the coupling between CBF (or BFV) and systemic BP at different frequencies, we introduced an intrinsic multiscale pressure-flow analysis (IMPFA) that is based on theories of nonlinear dynamics without the assumption of linearity and nonstationarity. The IMPFA quantifies dynamic phase relationship between intrinsic BP and BFV oscillations at different frequencies. The analysis includes three steps: (i) decompose BP and BFV signals into multiple intrinsic oscillatory modes each within a narrow frequency band (Figure 1); (ii) identify matched individual BP and BFV cycles from all oscillatory modes (Figure 2); and (iii) calculate BFV-BP phase shift for each matched BFV-BP cycle, assign individual cycles to different frequency bins based on cycle length, and calculate mean BFV-BP phase shift in each frequency bin.

(i) The first step was fulfilled using the empirical mode decomposition (EMD) (see details in Text S4) [27], [28], which allows the decomposition of a complex nonstationary signal into multiple empirical modes with each mode representing a frequency-amplitude modulation in a narrow band (Figure 1). Unlike the Fourier transform, the EMD is a nonlinear adaptive decomposition processes without assuming the shapes of waveforms. Thus, the resultant BP (or BFV) components are true intrinsic oscillatory functions embedded in the complex fluctuations.

(ii) For each BP mode and its corresponding BFV mode, instantaneous BP and BFV phases at all time points were obtained using the Hilbert transform. Then the BP mode and the BFV mode were divided into individual cycles with each cycle corresponding to a phase increment of 360°, e.g., from 0° to 360° and from 360° to 720° (Figure 2). Not all EMD-derived BP and corresponding BFV cycles necessarily reflect true underlying BFV-BP interactions. This can be caused by influences of noise or artifacts in the recordings such as artifacts in BFV signals when subjects were talking or when head movements affected the insonation angle of the TCD probe, missing data during the calibration of Finapres device, and changes in BP signals due to finger movement. Thus, we introduced the following criteria to exclude BP-BFV cycles that were possibly contaminated by noise and artifacts:

**Criterion 1.** There was a decrease in instantaneous BP or BFV phases in a cycle (Figure 2B);
**Criterion 2.** There were more than 10 points (0.2 seconds) in a cycle with instantaneous frequencies (based on instantaneous phase changes) much larger than the mean frequency of the cycle (≥2.5 times);
**Criterion 3**. The BFV cycle length is much larger (≥1.5 times) or much smaller (≤1/1.5 time) than the corresponding BP cycle length (Figure 2B).

The target of Criteria 1 and 2 is BFV or BP cycles that contain extra oscillations at higher frequencies while Criterion 3 is aimed for BFV (or BP) changes that were unrelated to BP (BFV) changes. For all matched BFV-BP cycles we used for the estimation of BFV-BP phase shifts, the difference in frequencies based on BP and BFV was 0.00067±0.0002 Hz (SE).

(iii) For each matched BFV-BP cycle, the start and end points of the cycle were based on the determined BP cycle, and phase shift was calculated by averaging all instantaneous phase differences between BFV and BP components in the cycle. All matched BFV-BP cycles were pooled and divided into non-overlapped frequency bins based on cycle length. There were 18 frequency bins with size of 0.02 Hz that cover the frequency range from 0.02 Hz to 0.38 Hz. Mean BFV-BP phase shift in each frequency bin was calculated from all cycles in the bin.

### Statistical analysis

Descriptive statistics were used to summarize data. One-way analysis of variance was used for between-group comparisons of age, body mass index, mean heart rate, and mean BP, mean BFV, CO_2_, cerebral resistance, and CO_2_ vasoreactivity. To assess the effects of frequency, group and their interaction on BFV-BP phase while accounting for possibly different or missing data points in certain bin(s) for different subjects, a mixed model ANOVA with subject nested in group as a random factor was performed (JMP-9.0 SAS Institute, Cary, NC). A similar mixed model was used to assess the potential difference between stroke and non-stroke sides in the stroke patients. In addition, possible influences on BFV-BP phase shift of age, sex, BMI, heart rate, mean BP, CO_2_, cerebral resistance, CO_2_ vasoreactivity were also explored using the mixed model.

## Supporting Information

Figure S1 **Variations of frequency in a BP oscillatory component.** (**A**) Blood pressure recording of a non-stroke subject. (**B**) One empirical mode extracted from the BP signal in A. There are 78 cycles in the ∼300 seconds. (**C**) Frequency of individual cycles.
(PDF)

Figure S2 **Phase shift due to time delay between BFV and BP recordings.** Shown are apparent BFV-BP phase shifts for different time delays, Δt, from −0.1 to 0.3 seconds (blue lines from bottom to top). The group means of BFV-BP phase shifts (Figure 3) and their polynomial fits were also plotted for comparison.
(PDF)

Figure S3 **Blood flow velocity in radial artery and middle cerebral artery simultaneously recorded from a non-stroke subject.** In each heartbeat, the peaks of two BFV signals were very close, i.e. time lag <50 ms.
(PDF)

Figure S4 **Coherence and BFV-BP phase shift derived from transfer function analysis (TFA).** (**A–B**) Coherence between BP and BFV in the left or non-stroke side (A) and the right or stroke side (B). Coherence <0.5 (gray region) indicates that the assumption of linearity is not valid. (**C–D**) TFA derived phase shift between BP and BFV in the left or non-stroke side (C) and the right or stroke side (D). Data are presented as Mean±SE, where Mean was obtained by averaging the individual means for non-stroke (or left) and right (stroke) sides and SE indicates between-subject error. Only data points with coherence >0.5 were included for the analysis of TFA phase. Shown are P values for the effects of group, frequency, and the interaction between group and frequency on TFA phase shift. Here “NS” indicates P>0.1.
(PDF)

Text S1 **Variations in BFV-BP phase shift due to nonstationary oscillations.**
(PDF)

Text S2 **Effects of time delay between BFV and BP recordings on phase shift estimation.**
(PDF)

Text S3 **Comparison of BFV-BP phase shifts derived from transfer function analysis and intrinsic multiscale pressure-flow analysis.**
(PDF)

Text S4 **Empirical mode decomposition.**
(PDF)
