# Supplementary figures and images for: A Nonlinear Dynamic Approach Reveals a Long-Term Stroke Effect on Cerebral Blood Flow Regulation at Multiple Time Scales
Source: PLoS Comput Biol. 2012 Jul 12;8(7):e1002601. doi: 10.1371/journal.pcbi.1002601 (PMC3395609; doi:10.1371/journal.pcbi.1002601)

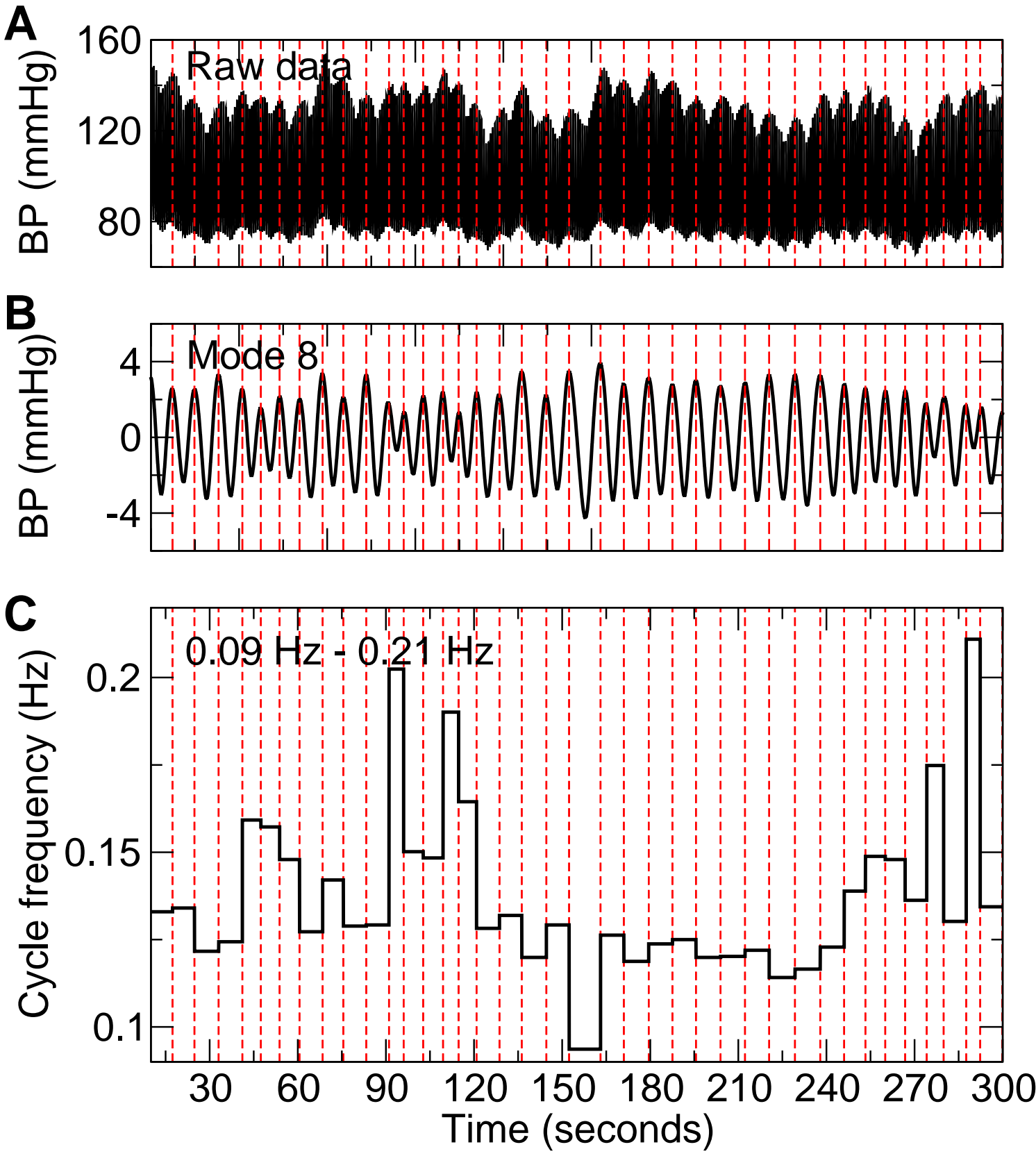

Supplement: Figure S1 — Variations of frequency in a BP oscillatory component. (A) Blood pressure recording of a non-stroke subject. (B) One empirical mode extracted from the BP signal in A. There are 78 cycles in the ∼300 seconds. (C) Frequency of individual cycles. (PDF) [file pcbi.1002601.s001.pdf]

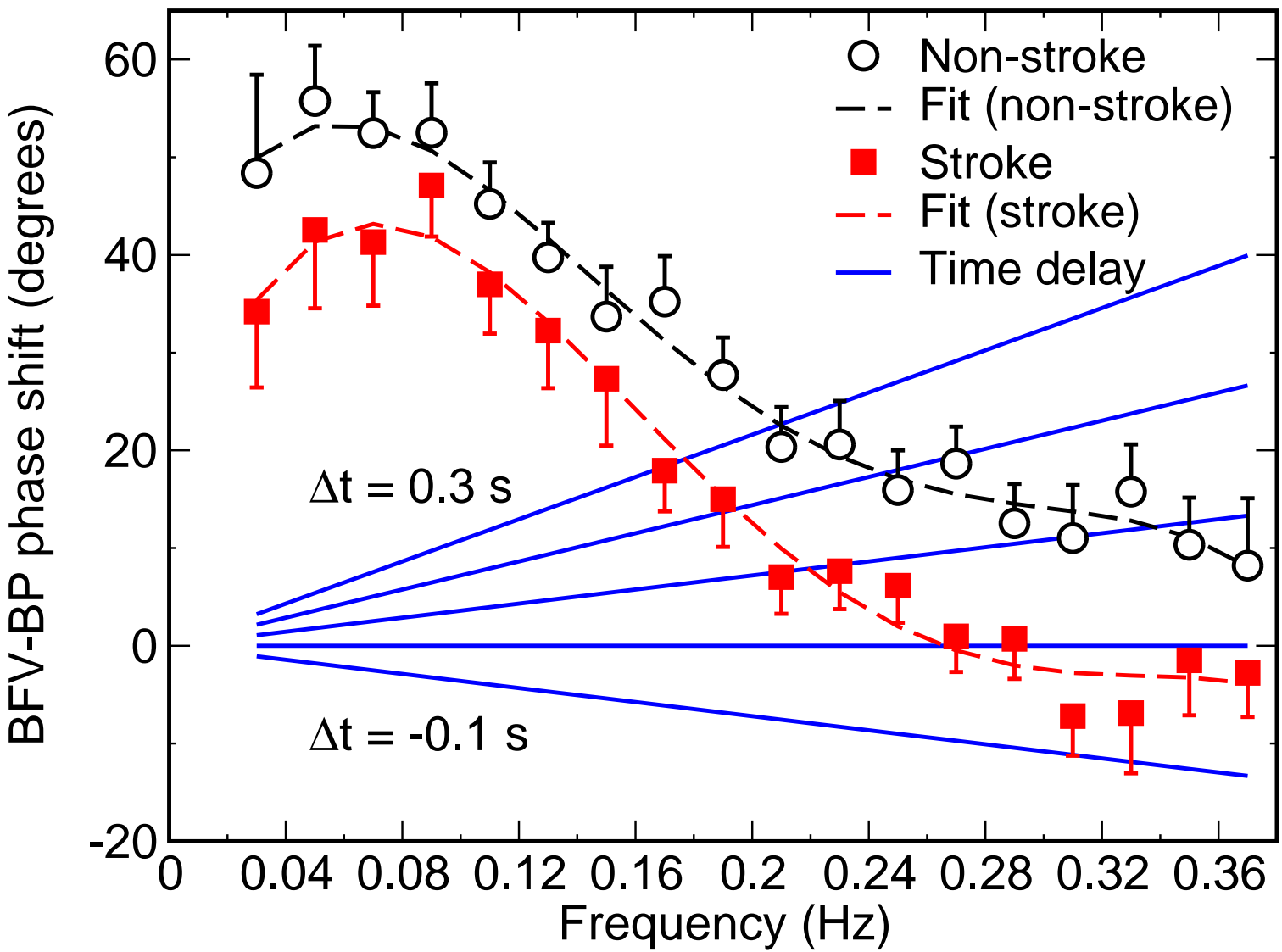

Supplement: Figure S2 — Phase shift due to time delay between BFV and BP recordings. Shown are apparent BFV-BP phase shifts for different time delays, Δt, from −0.1 to 0.3 seconds (blue lines from bottom to top). The group means of BFV-BP phase shifts (Figure 3) and their polynomial fits were also plotted for comparison. (PDF) [file pcbi.1002601.s002.pdf]

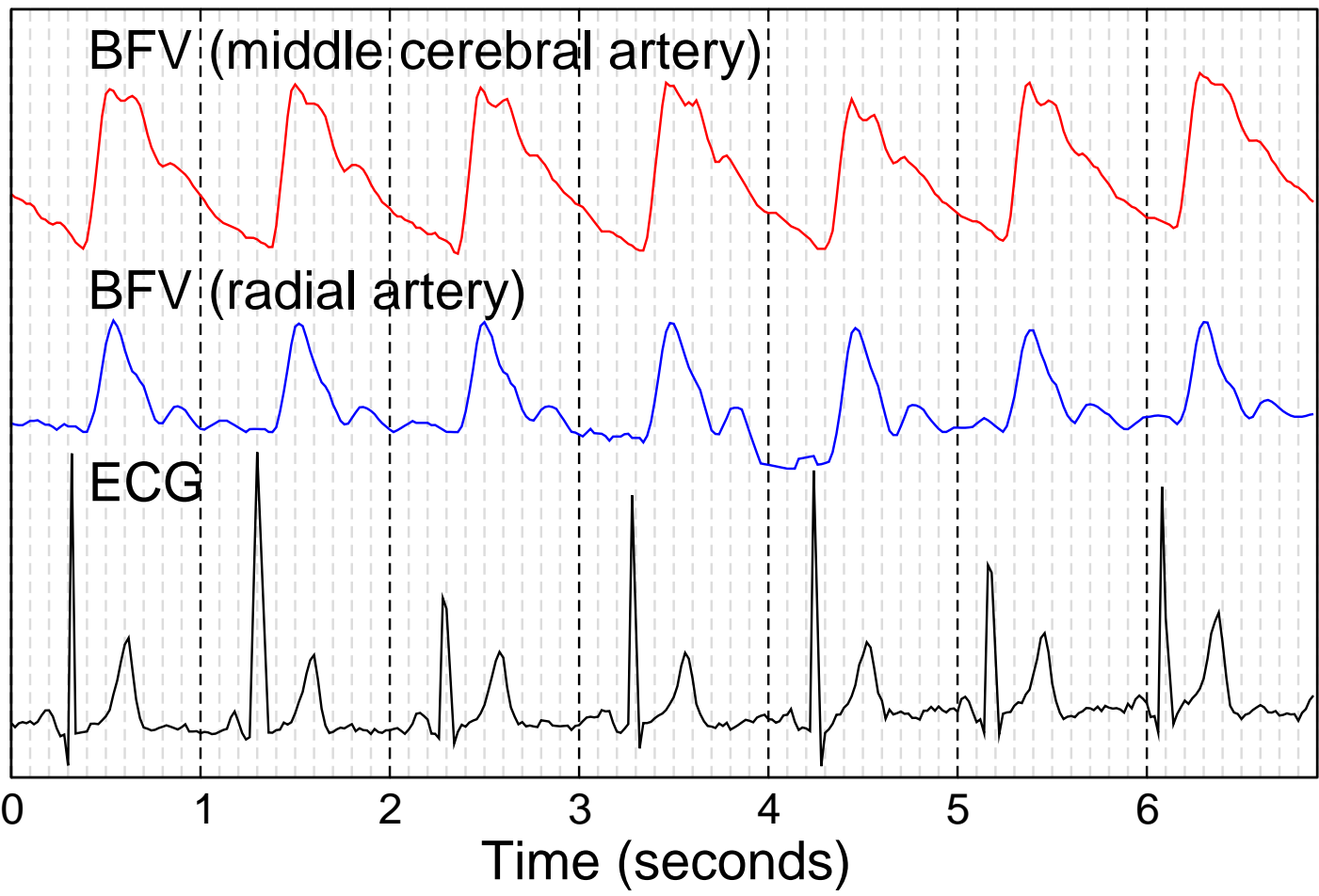

Supplement: Figure S3 — Blood flow velocity in radial artery and middle cerebral artery simultaneously recorded from a non-stroke subject. In each heartbeat, the peaks of two BFV signals were very close, i.e. time lag <50 ms. (PDF) [file pcbi.1002601.s003.pdf]

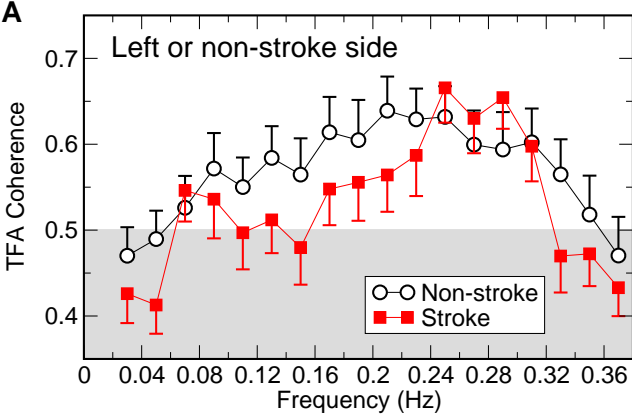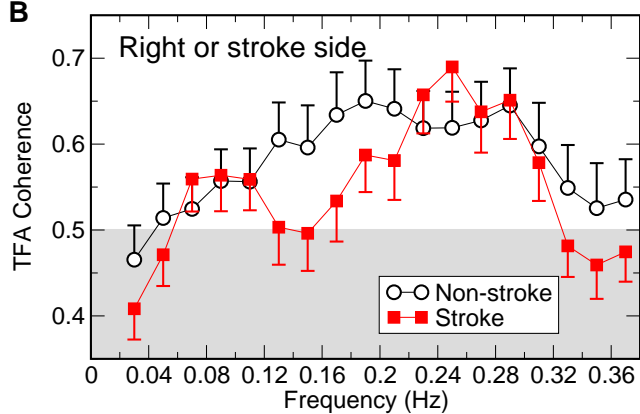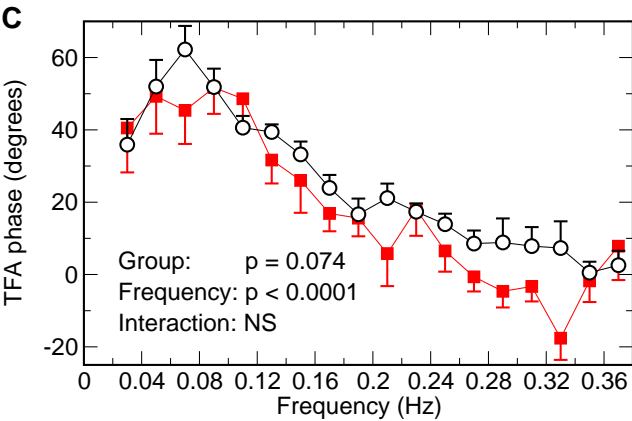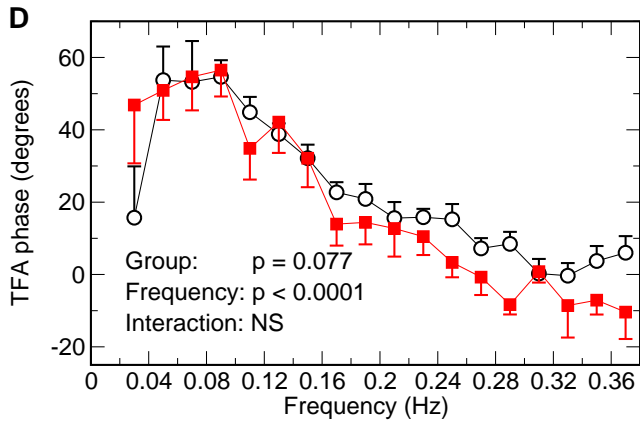

Supplement: Figure S4 — Coherence and BFV-BP phase shift derived from transfer function analysis (TFA). (A–B) Coherence between BP and BFV in the left or non-stroke side (A) and the right or stroke side (B). Coherence <0.5 (gray region) indicates that the assumption of linearity is not valid. (C–D) TFA derived phase shift between BP and BFV in the left or non-stroke side (C) and the right or stroke side (D). Data are presented as Mean±SE, where Mean was obtained by averaging the individual means for non-stroke (or left) and right (stroke) sides and SE indicates between-subject error. Only data points with coherence >0.5 were included for the analysis of TFA phase. Shown are P values for the effects of group, frequency, and the interaction between group and frequency on TFA phase shift. Here “NS” indicates P>0.1. (PDF) [file pcbi.1002601.s004.pdf]
