# Supplementary material for: A Nonlinear Dynamic Approach Reveals a Long-Term Stroke Effect on Cerebral Blood Flow Regulation at Multiple Time Scales
Source: PLoS Comput Biol. 2012 Jul 12;8(7):e1002601. doi: 10.1371/journal.pcbi.1002601 (PMC3395609; doi:10.1371/journal.pcbi.1002601)
Supplement: Text S1 — Variations in BFV-BP phase shift due to nonstationary oscillations. (PDF) [file pcbi.1002601.s005.pdf]

Variations in BFV-BP phase shift due to nonstationary oscillations

BP (and BFV) oscillations are often nonstationary as characterized by different frequency and amplitude for different cycles even within one oscillatory mode. Figure S1 shows a BP recording with well pronounced oscillations at a mean frequency of ~0.13Hz (i.e. 39 cycles in ~300 seconds). Further examination of individual cycles indicated that cycle length or frequency was time-variant, changing cycle by cycle with the lowest frequency = 0.09 Hz and the highest frequency = 0.21 Hz (Figure S1 C). Over this frequency range, BFV-BP phase shift could be very different. For instance, the mean phase shift decreased from ~52° at 0.09Hz to ~20° at 0.21 Hz in the non-stroke group, and decreased from ~47° at 0.09Hz to ~7° at 0.21 Hz in the stroke group (Figure 3). Considering the dramatic difference over such a narrow frequency range, it is important to study individual BFV and BP cycles in order to ensure reliable assessment of the multiscale BFV-BP phase relationship.
