# Supplementary material for: A Nonlinear Dynamic Approach Reveals a Long-Term Stroke Effect on Cerebral Blood Flow Regulation at Multiple Time Scales
Source: PLoS Comput Biol. 2012 Jul 12;8(7):e1002601. doi: 10.1371/journal.pcbi.1002601 (PMC3395609; doi:10.1371/journal.pcbi.1002601)
Supplement: Text S2 — Effects of time delay between BFV and BP recordings on phase shift estimation. (PDF) [file pcbi.1002601.s006.pdf]

Potential effects of time delay between BFV and BP recordings on phase shift estimation

It is possible that a pulse wave arrives at middle cerebral arteries earlier than it arrives at peripheral arteries in finger where BP is estimated. Such a difference in the pulse transit time can lead to an advanced phase of BFV oscillations as compared to BP oscillations (Figure S2), which might potentially explain the observed small BFV-BP phase shift at high frequencies in non-stroke subjects. For instance, a time delay of 0.3 seconds can lead to a phase shift of ~23° at frequency = 0.21 Hz, which is comparable to the observed value in the non-stroke subjects at the same frequency (Mean±SE: 20.3 ± 4.1^{o}). However, unlike the relationship between BFV-BP phase shift and frequency in human data and in the Aaslid-Tiecks model (Figure 5), the magnitude of the phase shift due to a time delay is larger at higher frequencies. For instance, as compared to frequency =0.21 Hz, the same time delay of 0.3 seconds will result in a much larger phase shift at frequency = 0.31Hz (~33°). This phase shift is also much larger than the value observed in the non-stroke subjects (11.0 ± 5.4^{o}). Thus, the BFV-BP phase shift across multiple frequencies is unlikely caused only by the possible effect of a time delay between BFV and BP signals.

In this retrospective study, we could not accurately quantify the difference in the pulse transit time for middle cerebral artery and peripheral artery because cerebral perfusion pressure was not available. However, we have been able to examine BFV recordings in radial artery that were simultaneously measured with the middle cerebral BFV in 5 non-stroke subjects. Figure S3 showed a representative recording of radial artery BFV within a few heart beats. The peak of radial artery BFV in each heart beat occurred slightly earlier than the peak of cerebral BFV but the delay was <50 ms. The similar results were observed in all 5 individuals with the maximal delay between radial

and cerebral BFV peaks <100 ms. We note that the time difference between cerebral and radial BFV peaks may not preciously represent the time delay due to different pulse transit time because there are possibly certain phase shifts between BFV and BP for both cerebral and radial arteries. Nevertheless, even if we assume a time delay of 100 ms between cerebral BFV and radial artery BP, the significant influences on BFV-BP phase shift should be at frequencies >0.3Hz (induced phase shift = 5.5^{o} at 0.15Hz, and 11° at 0.3Hz) (Figure S2).
