# Supplementary material for: A Nonlinear Dynamic Approach Reveals a Long-Term Stroke Effect on Cerebral Blood Flow Regulation at Multiple Time Scales
Source: PLoS Comput Biol. 2012 Jul 12;8(7):e1002601. doi: 10.1371/journal.pcbi.1002601 (PMC3395609; doi:10.1371/journal.pcbi.1002601)
Supplement: Text S3 — Comparison of BFV-BP phase shifts derived from transfer function analysis and intrinsic multiscale pressure-flow analysis. (PDF) [file pcbi.1002601.s007.pdf]

Transfer function analysis

We also assessed BFV-BP coupling using the traditional Fourier-transform-based transfer function analysis (TFA). BP and BFV signals were first linearly detrended and divided into 5000-point (100-sec) segments with 50% overlap. The Fourier transform of BP ($S_P(f)$) and BFV ($S_V(f)$) were calculated for each segment with a spectral resolution of 0.01Hz (averaged in 0.02 Hz bins), and were used to calculated the transfer function

$$H(f) = \frac{S_P(f)S_V^*(f)}{\left|S_P(f)\right|^2} = G(f)e^{i\phi(f)}$$

where $S_V^*(f)$ is the conjugate of $S_V(f)$; $\left|S_P(f)\right|^2$ is the power spectrum density of BP; $G(f) = \left|H(f)\right|$ is the transfer function amplitude (gain); and $\phi(f)$ is the transfer function phase at a specific frequency *f*. The amplitude and the phase of the transfer function reflect the linear amplitude and time relationship between the two signals. The reliability of these assumed linear relationships can be evaluated by coherence between the changes in pressure and velocity that ranges from 0 to 1:

$$C(f) = \frac{\left|S_P(f)S_V^*(f)\right|^2}{\left|S_P(f)\right|^2\left|S_V(f)\right|^2}$$

The linear assumption of the TFA requires higher coherence, and only under such condition, the TFA estimates of BFV-BP relationship are reliable [1]. Figure S4 A-B show the group means of BFV-BP coherence across the frequency range of 0.02-0.38 Hz. For both stroke and nonstroke groups, the mean coherence was less than 0.5 at many frequency bins, e.g., 0.02-0.06 Hz and 0.32-0.38Hz for the stroke group and 0.02-0.04 Hz for the non-stroke group. Individual results were more variable, i.e., in each frequency bin, many individuals had coherence >0.5 while other individuals had

coherence <0.5. Low coherence (<0.5) indicates that the assumed linear condition in the TFA is violated and, thus, that TFA phase (and gain) should be unreliable [1]. Thus, we only consider data points with coherence > 0.5 for the analysis of TFA phase shift.

Traditional transfer function analysis cannot reveal the effect of stroke on BFV-BP phase shift

Using a mixed model ANOVA, we examined the effects of frequency, group and their interaction on TFA phase while accounting for possibly different or missing data points in certain bin(s) for different subjects. Similar to the IMPFA results, TFA phase also showed a significant dependence on frequency (Figure S4 C-D). For both stroke and non-stroke groups, TFA phase exhibited a maximum between 0.04Hz and 0.1Hz and the value generally decreased at higher frequencies ($p<0.0001$), e.g., the average TFA phase at 0.02-0.1Hz was 44.2 ± 7.4^{o} (SE) for the non-stroke group and 52.7 ± 5.0^{o} for the stroke group, and the value at 0.3-0.38Hz was reduced to 7.7 ± 5.8^{o} for the control and -3.5 ± 3.6^{o} (statistically indistinguishable from zero; Wilcoxon signed rank test $p>0.5$) for the stroke group. Unlike the IMPFA results, TFA phase did not showed significant group difference across all frequency bins ($p> 0.07$ for both sides). The similar result was obtained (i.e., no group difference; $p>0.2$ for both sides) when considering the data in the low frequency region of <0.1 Hz where cerebral autoregulation is believe to be mostly active. Moreover, the mixed model ANOVA showed no significant interaction between the group and frequency ($p>0.3$ for both sides).

Comparison of BFV-BP phase shifts derived from TFA and IMPFA

To understand the different results based on TFA and IMPFA, we compared the BFV-BP phase shifts obtained from the two methods using a matched pairs test with frequency as the grouping factor (JMP-9.0 SAS Institute, Cary, NC). We did the test for the left (or non-stroke) side and the right (or stroke) side, separately. The analysis confirmed a strong effect of frequency ($p<0.0001$) but showed not significant difference between the mean TFA phase and the IMPFA-derived BFV-BP phase shift for both groups and for both sides (all p values > 0.3). These results suggested that there might be no systematic overestimation or underestimation of BFV-BP phase shift in the two methods, e.g., both method revealed the similar frequency dependence. The inconsistent findings with regard to the group difference should be mainly caused by influences of noise and nonstationarities that affect the estimation of BFV-BP phase shift [2]. It is possible that noise and nonstationarities induced more random errors in the TFA results as compared to the IMPFA results so that there was not enough power for the TFA to detect the subtle group using the small sample size of subjects.

References

1. Marmarelis VZ (1988) Coherence and apparent transfer function measurements for nonlinear physiological systems. Ann Biomed Eng 16: 143-157.

2. Lo MT, Novak V, Peng CK, Liu Y, Hu K (2009) Nonlinear phase interaction between nonstationary signals: a comparison study of methods based on Hilbert-Huang and Fourier transforms. Phys Rev E Stat Nonlin Soft Matter Phys 79: 061924.
