# Supplementary material for: A Nonlinear Dynamic Approach Reveals a Long-Term Stroke Effect on Cerebral Blood Flow Regulation at Multiple Time Scales
Source: PLoS Comput Biol. 2012 Jul 12;8(7):e1002601. doi: 10.1371/journal.pcbi.1002601 (PMC3395609; doi:10.1371/journal.pcbi.1002601)
Supplement: Text S4 — Empirical mode decomposition. (PDF) [file pcbi.1002601.s008.pdf]

Empirical mode decomposition

To obtain "true" oscillations in a BP or BFV signal at different frequencies, the intrinsic multiscale pressure-flow analysis (IMPFA) utilizes the empirical mode decomposition (EMD) algorithm based on Hilbert-Huang transform [1]. Each of the resulting oscillatory components, called intrinsic mode function (IMF), represents a frequency-amplitude modulation in a narrow band. Unlike the sinusoidal components derived from Fourier transform, the amplitude and period in each IMF can vary with time. Thus, the IMF can represent nonstationary oscillations embedded in physiological signals such as BP and BFV. The details of the EMD have been previously described [1]. Here we demonstrate the procedure of the EMD using a time series $x(t)$ that contains *n* IMFs:

$$\begin{aligned} x(t) &= c_1(t) + r_1(t) \\ &= c_1(t) + c_2(t) + r_2(t) \\ &\vdots \\ &= c_1(t) + c_2(t) + \cdots + c_n(t) \end{aligned} \tag{1}$$

Where $c_k(t)$ is the *k*-th IMF component and $r_k(t)$ is the residual after extracting the first *k* IMFs, i.e. $r_k(t) = x(t) - \sum_{i=1}^{k} c_i(t)$.

The EMD extracts IMFs one by one from the smallest scale to the largest scale using a sifting procedure. To extract the *k*-th IMF or $c_k(t)$, the sifting procedure includes the following steps:

(i) Assign $h_0(t) = h_{i-1}(t) = r_{k-1}(t)$ [for the first mode, k=1, $h_0(t) = x(t)$], where $i = 1$;

(ii) Extract local extrema of $h_{i-1}(t)$ (if there are less than 2 extrema, $c_k(t) = h_{i-1}(t)$ and stop the whole EMD process);

(iii) interpolate local minima of $h_{i-1}(t)$ to obtain the upper envelope function, $p(t)$, and interpolate local maxima of $h_{i-1}(t)$ to obtain lower envelope function, $v(t)$;

(iv) Calculate $h_i(t) = h_{i-1}(t) - \frac{p(t)+v(t)}{2}$, and the standard deviation (SD) of

$\frac{p(t)+v(t)}{2}$;

(v) If SD is small enough (less than a chosen threshold $SD\max$, typically between 0.2 and 0.3), the *k*-th IMF component is assigned as $c_k(t) = h_i(t)$ and $r_k(t) = r_{k-1}(t) - c_k(t)$; Otherwise repeat steps (ii) to (iv) for $i+1$ until $SD < SD\max$.

The above steps are repeated to obtain different IMFs at different scales until there are less than 2 minima or maxima in a residual $r_{n-1}(t)$ which is assigned as the last IMF $c_n(t)$ (see Step ii above).

References

1. Huang W, Shen Z, Huang NE, Fung YC (1998) Engineering analysis of biological variables: an example of blood pressure over 1 day. Proc Natl Acad Sci U S A 95: 4816-4821.
